# An efficient synthesis of tetramic acid derivatives with extended conjugation from *L*-Ascorbic Acid

**DOI:** 10.1186/1860-5397-2-24

**Published:** 2006-12-06

**Authors:** Biswajit K Singh, Surendra S Bisht, Rama P Tripathi

**Affiliations:** 1 Medicinal and Process Chemistry Division, Central Drug Research Institute, Lucknow-226001, India

## Abstract

**Background:**

Tetramic acids with polyenyl substituents are an important class of compounds in medicinal chemistry. Both solid and solution phase syntheses of such molecules have been reported recently. Thiolactomycin, a clinical candidate for treatment of tuberculosis has led to further explorations in this class. We have recently developed an efficient synthesis of tetramic acids derivatives from *L*- ascorbic acid. In continuation of this work, we have synthesised dienyl tetramic acid derivatives.

**Results:**

5,6-*O*-Isopropylidene-ascorbic acid on reaction with DBU led to the formation of tetronolactonyl allyl alcohol, which on oxidation with pyridinium chlorochromate gave the respective tetranolactonyl allylic aldehydes. Wittig olefination followed by reaction of the resulting tetranolactonyl dienyl esters with different amines resulted in the respective 5-hydroxy lactams. Subsequent dehydration of the hydroxy lactams with *p*-toluene sulphonic acid afforded the dienyl tetramic acid derivatives. All reactions were performed at ambient temperature and the yields are good.

**Conclusion:**

An efficient and practical method for the synthesis of dienyl tetramic acid derivatives from inexpensive and easily accessible ascorbic acid has been developed. The compounds bear structural similarities to the tetramic acid based polyenic antibiotics and thus this method offers a new and short route for the synthesis of tetramic acid derivatives of biological significance.

## 1. Background

**Figure 1:**
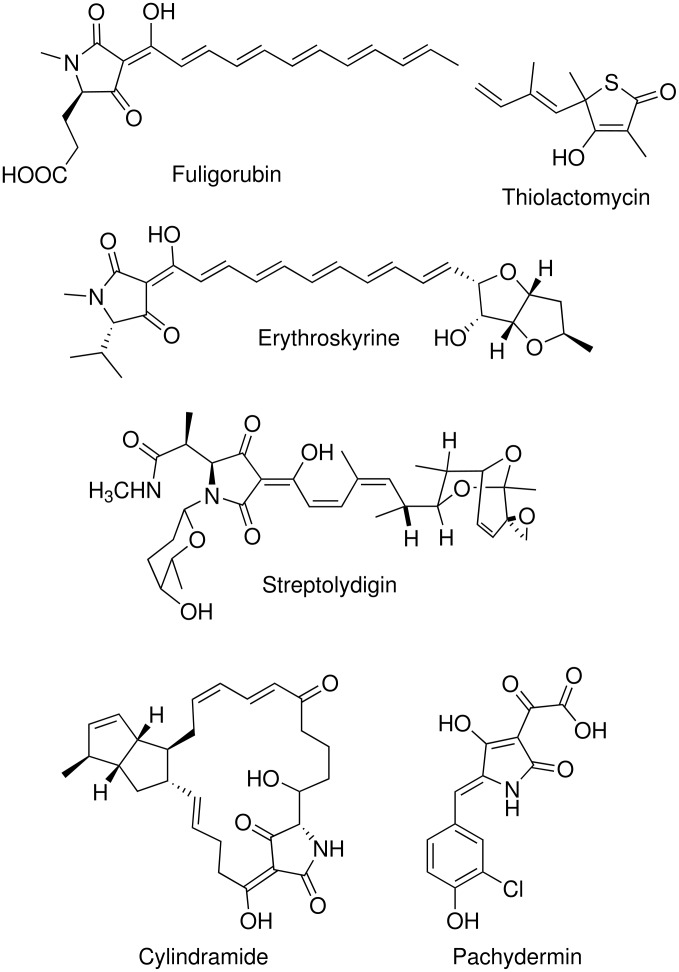
Tetramic acid antibiotics from natural sources.

Tetramic acid derivatives constitute an important class of nitrogen heterocycles with pyrrolidine-2,4-dione moieties and are key structural motifs in many natural products of terrestrial and marine origin. [1–6] They exhibit a wide range of biological activities including antibiotic, antiviral, antifungal, cytotoxic and enzyme inhibitory activities against bacterial DNA-directed RNA polymerases. [7–9] A few of the recently discovered tetramic acid antibiotics with dienyl or polyenyl units are shown in Figure 1. 3-Acetyl tetramic acid derivatives are known to act as anti-HSV and anti-HIV agents with potent tyrosine phosphatase inhibitory activities. [10–12] The distinct structural features and pharmacological properties of tetramic acid antibiotics pose challenges to organic chemists [13–14] to develop simple, practical and efficient syntheses of these compounds. Many groups are engaged in the synthesis of such molecules in sufficient quantities in order to study their *in vivo* activities and detailed modes of action. A variety of multi-step solid and solution phase syntheses exist for the preparation of both achiral and chiral tetramic acid derivatives. [15–31]

In an ongoing programme towards the development of new antitubercular agents from sugars, we have been interested in the synthesis of tetramic acid analogues bearing alkenyl chains at C-5. Our curiosity in this class was based on reports that similar structures, the thiolactomycins [32–33] (Figure 1), a class of thiotetronic acid with an alkenyl chain at C-5, possess mycobacterial FAS-II inhibitory activity and are potentially new tuberculosis drugs. 5-Alkenyl tetramic acids, being structurally similar to thiolactomycins, possess anti-HCV and anti-HIV activities [34–35] and are likely to yield new antitubercular prototype compounds active against tuberculosis in HIV cases. We have developed a one-pot synthesis of 5-hydroxyl tetramic acid derivatives without alkenyl substitutents at C-5, [36] but none of these compounds possesses significant activity against *Mycobacterium tuberculosis*. In continuation of this study tetramic acid derivatives with 5-alkenyl substitutents were synthesized starting from cheap and easily accessible ascorbic acid, commonly known as vitamin C.

## 2. Results and discussion

Ascorbic acid **1** was converted into 2,3-di-*O*-methyl and benzyl-5,6-*O*-isopropylidene ascorbic acid derivatives **3** and **4** via 5,6-*O*-isopropylidene ascorbic acid **2** following our modified earlier method. [36] These compounds were treated with DBU separately to get the intermediate allyl alcohols **5** and **6** in good yields, and the structures were confirmed by analysis of the spectroscopic data (Supporting Information File 1). The *Z* geometry of the double bond in these compounds has been established based on mechanistic grounds. [36–37]

**Scheme 1:**
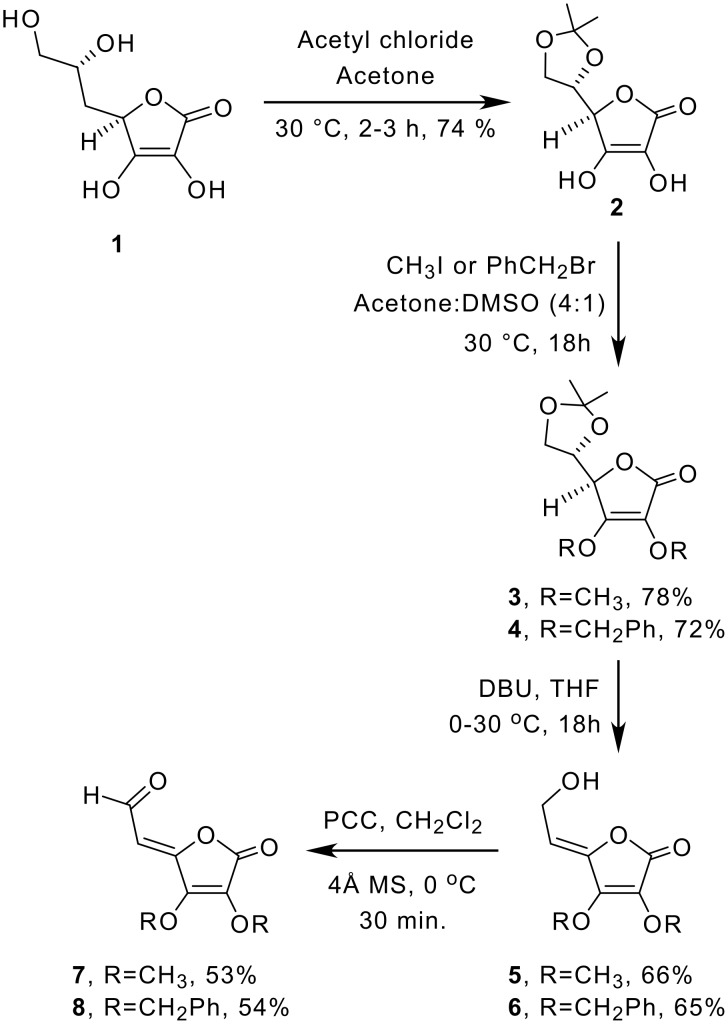
Synthesis of tetronolactonyl aldehydes from *L*-ascorbic acid

Pyrdinium chlorochromate oxidation of allylic alcohols **5** and **6** in dichloromethane, in the presence of molecular sieves (4 Å), led to the formation of tetronolactonyl allylic aldehydes **7** and **8** in good yields respectively (Scheme 1). The structures and geometry (*Z*) of these compounds were determined on the basis of their spectroscopic data (Supporting Information File 1).

**Scheme 2:**
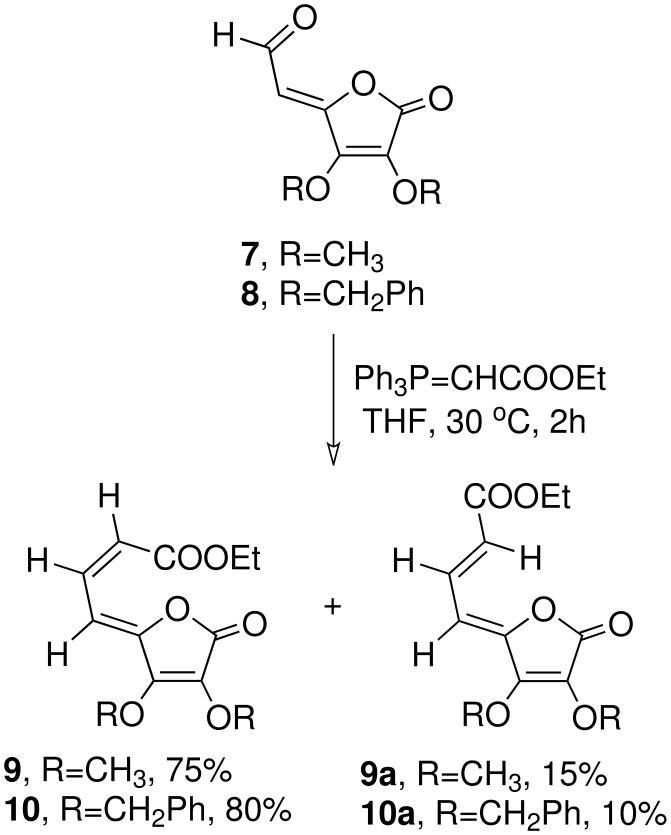
Synthesis of tetronolactonyl dienyl esters from etronolactonyl aldehydes

**Figure 2:**
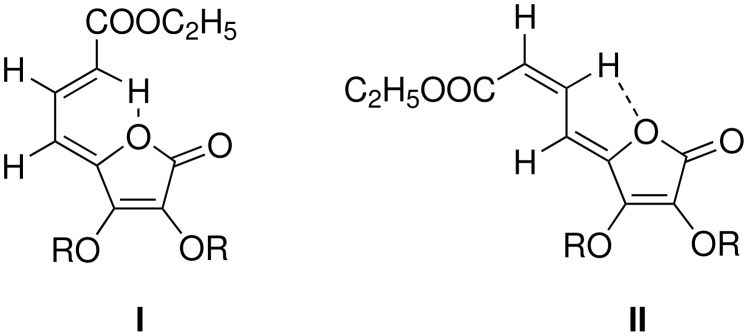
H-bonding in tetronolactonyl dienyl esters.

Wittig olefination of aldehydes **7** and **8** with carbethoxymethylene triphenylphosphorane in THF at ambient temperature led to the formation of the respective *exo*-dienyl esters of the tetronolactones as a mixture of *ZZ* and *ZE* isomers **9** and **9a** (17:3), and **10** and **10a** (9:1) respectively in quantitative yield (Scheme 2). The two isomers in each case were separated by column chromatography; the ratio and the structure of the individual isomers of the above compounds were determined on the basis of spectroscopic studies. In such an earlier study, [37] with BuLi at -78°C used as the base for the Wittig olefination, lower yields and poor stereoselection was observed as compared to our uncatalysed ambient temperature reaction where *ZZ* isomers were predominantly formed. The *Z* configuration of the allylic alcohol was already established. [36] The geometry of the newly generated double bond in **9** and **9a** was decided on the basis of ^1^H NMR spectroscopic data (Supporting Information File 1) wherein H-7 and H-8 in the major isomer **9** appeared as dd (δ = 7.57) with *J* = 11.9 Hz each and d, 5.96 (*J* = 11.9 Hz, H-7). The analogous protons in the minor isomer **9a** appeared as a dd δ = 7.0 (*J* = 11.3 Hz, 11.4 Hz, H-7) and δ = 7.27 (d, *J* = 11.1 Hz, 1H, H-8) respectively indicating the *Z* and *E* relationship of H-7 with H-8 in the above two isomers. The ^1^H NMR spectra of the *ZZ* and *ZE* isomers were similar to those reported earlier [37] and the chemical shifts of H-6 and H-8 were almost similar in the major isomer **9** in spite of the close proximity of H-8 with the carbethoxy group. The low chemical shift of H-8 in minor isomer **9a** may be due to its locked hydrogen bonded six member ring conformation. The low chemical shift of H-7 in the *ZZ* isomer may again be explained in terms of conformation II (Figure 2) as proposed by Khan *et al*, [37] where H-7 is hydrogen bonded to the lactone ring oxygen. Similarly, the structure and geometrical stereochemistry of the two isomers (**10** and **10a**) were also established.

The next step consists of reaction of the above dienyl tetronic acids with different amines separately to form the required intermediate 5-hydroxy tetramic acid derivatives. Thus reaction of **9** first with ethanolic ammonia led to the formation of 5-hydroxy lactam derivative **11** in good yield. The structure elucidation of compound **11** was based on its spectroscopic data and microanalysis (Supporting Information File 1). ESI MS of the compound showed [M+Na]^+1^ at 294.1 corresponding to its molecular formula. In the ^1^H NMR spectrum of compound **11**, H-6 appeared as a multiplet at δ 2.60–2.77 integrating for two protons, with H-7 at δ 6.81. The exchangeable C5-OH appeared as a singlet at δ 1.9, while H-8 appeared at its usual chemical shift of δ 5.87 as a doublet. The geometry of the double bond between C-7/C-8 was unaffected and it was Z only. Furthermore, we did not observe any conjugate addition product in the above reaction.

**Scheme 3:**
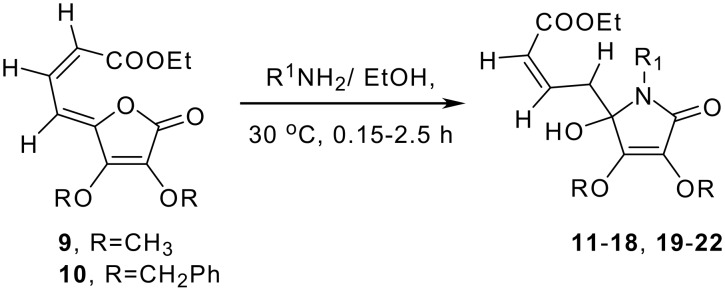
Synthesis of 5-hydroxy lactams from dienyl tetronic esters

Similarly, reaction of dienyl ester **9** with *n*-propyl-, cyclopropyl-, *n*-butyl-, *iso*-butyl-, *n*-hexyl-, *n*-octyl- and benzyl amines separately led to the formation of the respective *N*-alkyl lactams (**12–18**) in good to very good yields. However, reaction of compound **9**, bearing a 3, 4-dibenzyloxy substituent, with selected amines, *viz*. *n*-butyl-, *n-*hexyl and benzyl amines separately under the identical experimental conditions, led to the formation of the respective 5-hydroxy lactam derivatives (**19–22**) in good yields (see Table 1).

**Table 1:** Synthesis of 5-hydroxy lactams (**11–22**) from dienyl tetronic acid derivatives

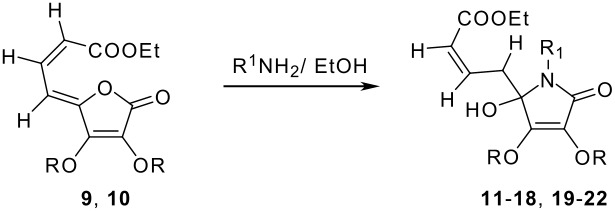				
Reactant	Product	R	R_1_	% Yield

**9**	**11**	-CH_3_	H	98
**9**	**12**	-CH_3_	-CH_2_Ph	73
**9**	**13**	-CH_3_	n-butyl	80
**9**	**14**	-CH_3_	cyclopropyl	80
**9**	**15**	-CH_3_	iso-butyl	65
**9**	**16**	-CH_3_	*n*-hexyl	77
**9**	**17**	-CH_3_	*n*-octyl	73
**9**	**18**	-CH_3_	*n*-propyl	81
**10**	**19**	-CH_2_Ph	H	86
**10**	**20**	-CH_2_Ph	*n*-butyl	74
**10**	**21**	-CH_2_Ph	*n*-hexyl	73
**10**	**22**	-CH_2_Ph	-CH_2_Ph	82

**Scheme 4:**
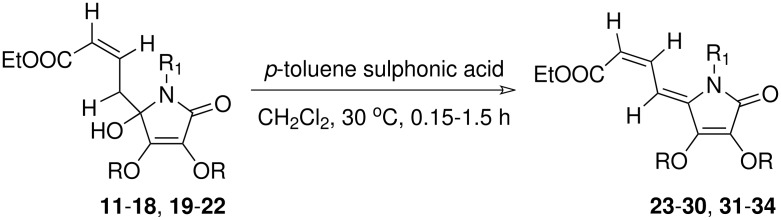
Synthesis of dienyl tetramic acid from 5- hydroxy lactams

Finally, the hydroxy lactams so obtained were dehydrated to the respective dienyl tetramic acid derivatives with *p*-toluenesulphonic acid (*p*-TSA) catalysed reaction at ambient temperature (Scheme 4). Thus, reaction of the above 5-hydroxy lactam **11** with *p*-TSA in CH_2_Cl_2_ at room temperature led to the formation of dienyl tetramic acid **23** in quantitative yield. The *ZZ* geometry of the two double bonds in compound **23** was established based on the basis of its ^1^H NMR spectrum (Supporting Information File 1).

Similarly, other dienyl tetramic acid derivatives **24–30** were prepared simply by dehydration of the respective hydroxyl lactams **12–18** in good yields. Similar dehydration of the hydroxy lactam derivatives **19–22** with *p*TSA in CH_2_Cl_2_ led to the formation of dienyl lactam derivatives **31–34** in good yields (see Table 2).

**Table 2:** Synthesis of dienyl tetramic acid derivatives from 5-hydroxy lactams

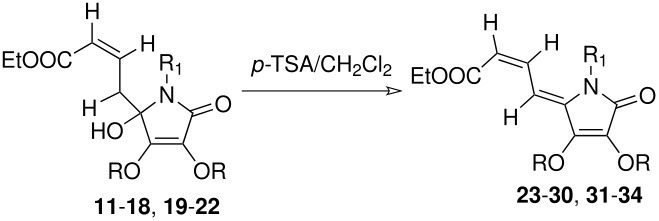				
Reactant	Product	R	R1	% Yield

**11**	**23**	-CH_3_	H	69
**12**	**24**	-CH_3_	-CH_2_Ph	65.5
**13**	**25**	-CH_3_	n-butyl	71
**14**	**26**	-CH_3_	cyclopropyl	62
**15**	**27**	-CH_3_	iso-butyl	58
**16**	**28**	-CH_3_	*n*-hexyl	55
**17**	**29**	-CH_3_	*n*-octyl	60.5
**18**	**30**	-CH_3_	*n*-propyl	62
**19**	**31**	-CH_2_Ph	H	63
**20**	**32**	-CH_2_Ph	*n*-butyl	55
**21**	**33**	-CH_2_Ph	*n*-hexyl	62
**22**	**34**	-CH_2_Ph	-CH_2_Ph	55

The structures of all the compounds were determined on the basis of spectroscopic data and microanalysis (Supporting Information File 1). Detailed experimental procedures for the preparation of compounds and full characterization data can be found in Supporting Information File 1.

## 3. Conclusion

In conclusion, we have developed an efficient and practical method for the synthesis of dienyl tetramic acid derivatives from inexpensive and easily accessible ascorbic acid. The method involves Wittig olefination of the allylic aldehydes obtained from ascorbic acid followed by reaction of the resulting esters with amines to give the intermediate 5-hydroxy lactams. The latter on dehydration with p-toluene sulphonic acid resulted in dienyl tetramic acid derivatives. The compounds bear structural similarities to the tetramic acid based polyenic antibiotics and the method paves the way for the synthesis of a variety of tetramic acid derivatives with different substitutents. Detailed bioevaluation of these compounds for different activities is under way.

## Supporting Information

File 1 supplementary information
