# Supplementary material for: An efficient synthesis of tetramic acid derivatives with extended conjugation from L-Ascorbic Acid
Source: Beilstein J Org Chem. 2006 Dec 6;2:24. doi: 10.1186/1860-5397-2-24 (PMC1783862; doi:10.1186/1860-5397-2-24)
Supplement: File 1 — supplementary information [file Beilstein_J_Org_Chem-02-24-s001.pdf]

An efficient synthesis of tetramic acid derivatives with extended conjugation from L-Ascorbic Acid[#]

Biswajit K. Singh, Surendra .S. Bisht and Rama P. Tripathi*

Medicinal and Process Chemistry Division, Central Drug Research Institute, Lucknow-226001

Experimental

Commercially available reagent grade chemicals were used as received. All reactions were followed by TLC on Merck Kieselgel 60 F_{254}, with detection by UV light and/or spraying 20% $KMnO_4$ agueous solution. Column chromatography was performed on Silica Gel (230–400 mesh, Merck). IR spectra were recorded as thin films or neat chloroform solution with a Perkin-Elmer Spectrum RX-1 (4000-450 cm^{-1}) spectrophotometer. The ^1H and ^{13}C NMR spectra were recorded on a Bruker DRX -300 in (*D*) chloroform, shift values in ppm relative to $SiMe_4$ as internal reference, unless otherwise stated; signals are reported as s (singlet), d (doublet), t (triplet), m (multiplet); *J* in Hz. Fast atom bombardment mass spectra (FABMS) were performed by the Mass Spectrometer Jeol SX-102(FAB). Elemental analyses were performed on a Perkin-Elmer 2400 II elemental analyzer. The optical rotations were measured in a 1.0 dm tube with a Rudolf Autopol III polarimeter in chloroform. Solvents were dried and stored over activated 4Å molecular sieve.

5,6-*O*-isopropylidene-L-Ascorbic acid (2):

To a magnetically stirred solution of ascorbic acid (30 g, 170.4 mmol) in acetone (120 mL), acetyl chloride (3 mL, 42.6 mmol) was added and reaction mixture stirred for 2-3 hrs at ambient temperature and kept in cold for 7-8 h. The reaction mixture was filtered and washed with cooled acetone. The crude solid thus obtained was dried (27 g, 73.7%); m.p. 195-198 ºC. The crude was recrystalised by acetone and hexane, the m.p.

of recrystalised product was around 206-208 ºC; IR (neat): 3021, 1754, 1658, 1217 cm^{-1}; ^1H NMR (200 MHz, $CDCl_3$+DMSO-d^6) δ = 1.33(s, 3H, CH_3), 1.36 (s, 3H, CH_3), 3.20 (bs, 1H, OH), 3.98-4.26 (m, 3H, OCH_2 & OCH), 4.53 (d, J = 4.0 Hz, 1H, CH), 8.30 (bs, 1H, OH); ^{13}C NMR (50 MHz, $CDCl_3$+DMSO-d^6) δ = 30.8, 31.2, 70.4, 76.3, 76.7, 114.7, 124.1, 156.3, 175.4; MS (ESI) 217 $(M+H)^+$.

2,3-Dimethoxy-5,6-*O*-isopropylidene-L-Ascorbic acid (3): To a magnetically stirred solution of compound **2** (25 g, 115.7 mmol) in acetone and DMSO (4:1), K_2CO_3 (32 g, 231.4 mmol) methyl iodide (14.9 mL, 231.4 mmol) was added drop wise. Tetrabutyl ammonium bromide (2.0 g) was added to the stirring reaction mixture and stirring continued for 12 hr. The reaction mixture was portioned between ethyl acetate (150 mL) and water (100 mL). The organic layer was dried (Na_2SO_4) and evaporated under reduced pressure to give a crude mass, which was chromatographed over silica gel (230-400 mesh) using a gradient of hexane-EtOAc (17:3) as eluent to give **3** as colourless solid (22 g, 78%); IR (neat): 1762, 1680 cm^{-1}; ^1H NMR (200 MHz, $CDCl_3$) δ = 1.36 (s, 3H, CH_3), 1.39 (s, 3H, CH_3), 3.85 (s, 3H, OCH_3), 4.0-4.10 (m, 2H, OCH_2), 4.16 (s, 3H, OCH_3), 4.25-4.52 (m, 2H, OCH & CH); ^{13}C NMR (50 MHz, $CDCl_3$)) δ = 25.9, 26.2, 59.8, 60.7, 65.6, 74.2, 74.8, 110.7, 123.5, 157.1, 169.3; MS (ESI) 245 $(M+H)^+$.

2,3-Dibenzyloxy-5,6-*O*-isopropylidene-L-Ascorbic acid (4): It was obtained by the reaction of **2** (25 g, 115.7 mmol), K_2CO_3 (32 g, 231.4 mmol) and benzyl bromide (27.4 mL, 231.4 mmol) using the above procedure. Colourless powder (33 g, 72%); IR (neat): 1764, 1679 cm^{-1}; ^1H NMR (200 MHz, $CDCl_3$) δ = 1.36 (s, 3H, CH_3), 1.40 (s, 3H, CH_3), 3.96-4.12 (m, 2H, OCH_2), 4.21-4.54 (m, 2H, OCH & CH), 5.03-5.21 (m, 4H, 2 x OCH_2Ph), 7.18-7.38 (m, 10H, ArH); ^{13}C NMR (50 MHz, $CDCl_3$) δ = 26.0, 26.3, 65.6, 73.9, 74.2, 74.3, 75.0, 109.9, 110.7, 121.5, 128.2 (3C), 129.0 (4C), 129.5 (3C), 135.7, 136.3, 157.0, 169.5; MS (ESI) 397.1 $(M+H)^+$.

(*Z*) – 3,4-Dimethoxy-5-(2-hydroxyethylidene)-5H-furan-2-one(5): ***General Procedure***

To a magnetically stirred solution of compound **3** 20 g (81.96 mmol) in THF (80 mL), DBU (6.2 mL, 50 mol %) was slowly added and the reaction mixture was stirred for 18 h at room temperature. The solvent was evaporated under reduced pressure and the residue, thus obtained, was dissolved in ethyl acetate (100mL), washed with water (25 mL). The organic layer was dried (Na_2SO_4) and evaporated under reduced pressure to afford a crude mass, which was chromatographed over silica gel (230-400 mesh) using a gradient of hexane-EtOAc (9:1→ 3:1) as eluent to give the above compounds as colourless solid (10 g, 65.7%), m.p 60 ºC, IR (Neat): 3391, 1688 cm^{-1}, ^1H NMR (200 MHz, $CDCl_3$) δ = 1.39 (bs, 1H), 3.92 (s, 3H, OCH_3), 4.16 (s, 3H, OCH_3), 4.41 (d, *J* = 7.0, 2H, OCH_2), 5.50 (t, *J* = 7.0 Hz, 1H, =CH); ^{13}C NMR (50 MHz, $CDCl_3$) δ = 56.6, 59.9, 60.6, 108.1, 125.0, 142.3, 149.1, 164.7; MS (FAB) 187$(M+H)^+$.

(Z) 3,4-Dibenzyloxy-5-(2-hydroxyethylidene)-5H-furan-2-one (6):.

It was obtained by reaction of **4** 20g (50.5 mmol) and DBU 3.9 mL (50 mol%) as described above. Colourless oil, yield (11 g, 64.7%); IR (neat): 3389, 1676 cm^{-1}; ^1H NMR (200 MHz, $CDCl_3$) δ = 1.42 (bs, 1H), 4.40 (d, *J* = 7.0 Hz, 2H, OCH_2), 5.16 (s, 2H, OCH_2Ph), 5.22 (s, 2H, OCH_2Ph), 5.51 (t, *J* = 7.0 Hz, 1H, =CH), 7.21- 7.37(m, 10H, ArH); ^{13}C NMR (50 MHz, $CDCl_3$) δ = 56.9, 73.6, 74.4, 108.0, 124.1, 128.0 (2C), 129.0 (2C), 129.1 (3C), 129.3 (3C), 135.8, 136.1, 142.7, 148.7, 167.5; MS (FAB) 339 $(M+H)^+$.

(*Z*) (3, 4-Dimethoxy-5-oxo-*5H*-furan-2-ylidene)-acetaldehyde (7): General Procedure

To a magnetically stirred mixture of powdered dried molecular sieve (4Å, 10 g) and PCC (11.6 g, 51.6 mmol) in dry CH_2Cl_2 (120 mL), a solution of the above allyl alcohol **5** (8.00 g, 43.0 mmol) in CH_2Cl_2 (20 mL) was added drop wise at 0 °C and stirring continued for half an hour. The reaction mixture was filtered over a pad of celite and celite cake was washed with more dichloromethane. The filtrate was evaporated to yield a crude mass which was purified by flash chromatography using a gradient of hexane-EtOAc (9:1→ 4:1) to give compound 2a as a light yellow solid, m.p 61-63 ºC; Yield (4.20 g, 53%); IR (neat) 1788, 1656 cm^{-1}; ^1H NMR (200 MHz, $CDCl_3$) δ = 4.05 (3H, s,

OCH_3), 4.18 (3H, s, OCH_3), 5.65 (1H, d, *J* = 8 Hz, =CH), 10.08 (1H, d, *J* = 8Hz, CH=O); ^{13}C NMR (50 MHz, $CDCl_3$) δ = 60.2, 60.5, 105.0, 126.5, 147.2, 155.9, 162.4, 188.1; MS (ESI) 207 $(M+Na)^+$.

(*Z*)–(3, 4-Dibenzyloxy-5-oxo-5H-furan-2-ylidene)-acetaldehyde (8)

It was obtained from compound **6** (9.00 g, 26.6 mmol) as above with a mixture of powdered dried molecular sieve (4Å,11 g) and PCC (6.3 g, 31.9 mmol) and in dry CH_2Cl_2 (120 mL) as a light yellow solid (5.9 g, 54%); IR (neat): 1790, 1656 cm^{-1}; ^1H NMR (200 MHz, $CDCl_3$) δ = 5.27 (2H, s, OCH_2Ph), 5.30 (2H, s, OCH_2Ph), 5.67 (1H, d, *J* = 8 Hz, =CH), 7.21-7.37 (m, 10H, ArH),10.06 (1H, d, *J* = 8Hz, CH=O); MS (ESI): 337 $(M+H)^+$.

4-(3, 4-Dimethoxy-5-oxo-*5H*-furan-2-ylidene)-but-2-enoic acid ethyl ester (9 and 9a): General Procedure:

A mixture of the above aldehyde **7** (4.0 g, 21.73 mmol) and carbethoxymethylenetriphenyl phosphorane (8.3 g, 23.9 mmol) in *anh*. THF (25 mL) was magnetically stirred for 1.5 h. The solvent was evaporated and the residue was dissolved in ethyl acetate (100 mL) and washed with water (2 x 25 mL), organic layer was dried (Na_2SO_4) and evaporated under reduced pressure to give a crude mass. The latter on column chromatography over silica gel (60-120 mess) using a gradient of hexane: EtOAc (19:1→ 7:1) as eluent gave the above compound as a (*Z,Z*) **9** and (*Z,E*) **9a** isomers in the ratio of 17:3;

Z,Z-isomer (**9**): IR (neat): 1780, 1596 cm^{-1}; ^1H NMR(200MHz, $CDCl_3$) δ = 1.31(t, *J* = 7.1 Hz, 3H, CH_3), 3.98 (s, 3H, OCH_3) and 4.15 (s, 3H, OCH_3), 4.20 (q, *J* = 7.1 Hz, 2H, OCH_2), 5.91(d, *J* = 11.9 Hz, 1H, 6-H), 5.96 (d, *J* = 11.9 Hz, 1H, H-8), 7.57 (dd, *J* = 11.9Hz each, 1H, H-7); ^{13}C NMR (50 MHz, $CDCl_3$): δ 14.6, 59.8, 60.6, 61.0, 105.3, 124.4, 126.2, 135.4, 146.0, 148.2, 163.6, 166.6; MS (FAB): 255 $(M+H)^+$.

Z,E-isomer (**9a**): Colourless solid, m.p. 65 ºC; IR (neat): 1777, 1707, 1602 cm^{-1}; ^1H NMR (300 MHz, $CDCl_3$) δ = 1.32 (t ,3H, J= 7.1 Hz, CH_3), 3.99 (s, 3H, OCH_3), 4.16 (s,

3H, OCH_3), 4.22 (q, 2H, *J* = 7.1 Hz, OCH_2), 5.82 (d, *J* = 11.2 Hz, 1H, H-6), 7.0 (dd, *J* = 11.4 Hz, 11.3 Hz, 1H, H-7), 7.27 (d, *J* = 11.1 Hz, 1H, H-8); ^{13}C NMR (50 MHz, $CDCl_3$) δ = 14.6, 59.6, 60.4, 73.0, 73.2, 103.4, 120.4, 126.2, 132.4, 134.7, 146.3, 148.5, 163.3, 166.3; MS (FAB): 255 $(M+H)^+$.

4-(3, 4-Dibenzyloxy-5-oxo-5H-furan-2-ylidene)-but-2-enoic acid ethyl ester (10 and 10a):

It was obtained by reaction of vinylic aldehyde **8** (4.5 g, 13.4 mmol) and carbethoxymethylene triphenyl phosphorane (5.1 g, 23.9 mmol) as above and purified by column chromatography over silica gel (60-120 mess) using a gradient of hexane:EtOAc (19:1→ 7:1) as eluent to give the above compounds as a (Z,Z) and (Z,E) isomer in the ratio of 9:1;

***Z,Z*-isomer (10)** : IR (neat) 1778, 1711, 1651 cm^{-1}; ^1H NMR(200 MHz, $CDCl_3$) δ = 1.30 (t, *J* = 7.0 Hz, 3H, CH_3), 4.22 (t, *J* = 7.0 Hz, 2H, CH_2), 5.23 (s, 4H, 2xOCH_2), 5.89 (d, *J* = 11.9 Hz, 1H, 6-H), 5.99 (d, *J* = 11.9 Hz, 1H, 8-H), 7.20-7.36 (m, 10H, ArH), 7.55(dd, *J* = 11.9 Hz each,1H, 7-H); MS (ESI): 429 (M+ Na$)^+$; ^{13}C NMR (50 MHz, $CDCl_3$) δ = 14.6, 61.1, 73.8, 74.4, 105.5, 124.5, 127.4, 128.2 (2C), 128.7, 129.1 (3C), 129.3 (3C); MS (ESI): 429 (M+ Na$)^+$.

***Z, E*-isomer (10a)**: solid, m.p. 55 ºC ; IR (neat): 1775, 1710, 1648 cm^{-1}; ^1H NMR(200 MHz, $CDCl_3$) δ = 1.27 (t, *J* = 7.1 Hz, 3H, CH_3), 4.12 (q, *J* = 7.1 Hz, 2H, OCH_2), 5.19 (s, 2H, OCH_2), 5.26 (s, 2H, OCH_2), 5.79 (d, *J* = 11.1 Hz, 1H, 6-H), 7.03 (t, *J* = 11.1 Hz, 1H, H-7), 7.22- 7.37 (m, 11H, H-8, ArH); ^{13}C NMR (50 MHz, $CDCl_3$) δ = 14.6, 60.5, 73.5, 74.2, 103.7, 120.6, 124.8, 127.9(2C), 128.1, 129.0, 129.1 (3C), 129.2 (3C), 134.7, 135.8, 136.0, 146.6, 148.2, 163.6, 166.3; MS (ESI): 429.1 (M+ Na$)^+$.

(*Z*) 4-(2-Hydroxy-3,4-dimethoxy-5-oxo-2,5-dihydro-1*H*-pyrrol-2-yl)-but-2-enoic acid ethyl ester (11): General Procedure:

A solution of the above compound **9** (1 g, 3.93 mmol) in ethanolic ammonia (15 mL) was magnetically stirred for 15 min. in a sealed vessel. The excess of ammonia and solvent were evaporated under reduced pressure to give a residual mass. The latter was

chromatographed over silica gel (60-120 mess) column using a gradient of hexane:EtoAc (2:3) to give **11** as, colourless foam (1 g, 98%); IR (neat): 3431, 1684,1564 cm^{-1}; ^1H NMR (200 MHz, $CDCl_3$) δ = 1.27 (t, *J* =7.1Hz, 3H, CH_3), 1.85(s,1H, OH), 2.60-2.77(m, 2H, CH_2), 3.77 (s, 3H, OCH_3), 4.09 (s, 3H, OCH_3), 4.15 (q, *J* =7.1 Hz, 3H, OCH_2CH_3), 5.87 (d, *J* = 15.6Hz, 1H, H-8), 6.81 (m, 2H, H-7 & -NH); ^{13}C NMR (50 MHz, $CDCl_3$) δ = 14.5, 39.4, 59.4, 60.7, 61.3, 83.3, 96.5, 125.6, 141.8, 155.0, 166.5, 170.2; MS (ESI): 294.1 $(M+Na)^+$; Anal. Calcd for $C_{12}H_{17}NO_6$: C, 53.13; H, 6.32; N, 5.16, Found: C, 53.15; H, 6.35; N, 5.14

(*Z*) 4-(1-Benzyl-2-hydroxy-3,4-dimethoxy-5-oxo-2,5-dihydro-1*H*-pyrrol-2-yl)-but-2-enoic acid ethyl ester (12): A solution of compound **9** (0.8 g, 3.14 mmol) in ethanol and benzyl amine (0.35 mL, 3.14 mmol) was magnetically stirred for 2.5 hrs. Solvent was evaporated under reduced pressure and the residue, thus obtained was dissolved in ethyl acetate (25 mL) and washed with water (10 mL). The organic layer was dried (Na_2SO_4) evaporated under reduced pressure to give crude mass which was purified by column chromatography over silica gel (60-120 mess) using a gradient of hexane:EtoAc (1:4) to give **12** as light brown solid, (0.82 g, 72.5%); m. p. 82-84 ºC; IR (neat): 3387,1674, 1598 cm^{-1}; ^1H NMR (200 MHz, $CDCl_3$) δ = 1.24 (t, *J* = 7.0 Hz, 3H, CH_3), 1.58 (s, 1H, OH), 2.52 (m, 2H, H-6), 3.82 (s, 3H, OCH_3), 4.06 (s, 3H, OCH_3), 4.11 (q, *J* = 7.0 Hz, 2H, OCH_2), 4.28(d, *J* = 15.2 Hz, 2H, NCH_APh), 4.65 (d, *J* = 15.2 Hz, 2H, NCH_BPh), 5.32 (d, *J* = 15.4 Hz, 1H, H-8), 6.15-6.23 (m,1H, H-7), 7.25-7.32 (m, 5H, ArH); ^{13}C NMR (50 MHz, $CDCl_3$) δ = 14.6, 37.3, 41.3, 59.4, 60.4, 60.9, 61.1, 86.8, 125.11, 127.8, 128.3, 128.4, 129.0, 138.7, 140.6, 153.0, 166.0, 168.5; MS (ESI): 384.1 $(M+Na)^+$; Anal. Calcd for $C_{19}H_{23}NO_6$: C, 63.15; H, 6.41; N, 3.88, Found: C, 63.12; H, 6.40; N, 3.90.

(*Z*) 4-(1-Butyl-2-hydroxy-3,4-dimethoxy-5-oxo-2,5-dihydro-1*H*-pyrrol-2-yl)-but-2-enoic acid ethyl ester (13): It was obtained by the reaction of compound **9** (0.9 g, 3.54 mmol) and butyl amine (0.26 mL, 3.54 mmol) using the above procedure as an oil (0.92 g, 79.8%); IR (neat): 3333, 1684 cm^{-1}; ^1H NMR (200 MHz, $CDCl_3$) δ = 0.93 (t, *J* =7.1 Hz, 3H), 1.27 (t, *J* =7.1 Hz, 3H), 1.55-1.65 (m, 4H, 2 x CH_2), 2.72-2.75 (m, 2H, CH_2),

3.01-3.40 (m, 2H, NCH_2), 3.40 (s,1H), 3.78 (s, 3H, OCH_3), 4.06 (s, 3H, OCH_3), 4.18 (q, *J* = 7.1 Hz, 2H, OCH_2), 5.82 (d, *J* = 15.6 Hz, 1H, H-8), 6.49-6.57 (m, 1H, H-7); ^{13}C NMR (50 MHz, $CDCl_3$) δ = 14.1, 14.6, 20.9, 32.0, 37.2, 38.2, 59.3, 60.6, 60.9, 86.3, 108.2, 125.3, 125.9, 141.1, 152.2, 166.0, 168.0; MS (ESI): 350.1 $(M+Na)^+$; Anal. Calcd for $C_{16}H_{25}NO_6$: C, 58.70; H, 7.70; N, 4.28, Found: C, 58.67; H, 7.72; N, 4.26.

(*Z*) 4-(1-Cyclopropyl-2-hydroxy-3,4-dimethoxy-5-oxo-2,5-dihydro-1*H*-pyrrol-2-yl)-but-2-enoic acid ethyl ester (14): It was obtained by the reaction of **9** (0.5 g, 1.96 mmol) and cyclopropyl amine (0.2 mL, 1.96 mmol) as an oil (0.43 g, 79.8%); IR (neat): 3419, 1688, 1596 cm^{-1}; ^{1}H NMR (200 MHz, $CDCl_3$) δ = 0.65-0.88 (m, 4H), 1.27 (t, *J* =7.1 Hz, 3H), 2.31-2.36 (m, 1H), 2.83-2.95 (m, 2H), 3.11 (s, 1H), 3.78 (s, 3H), 4.07 (s, 3H), 4.18 (q, *J* =7.1 Hz, 2H), 5.85 (d, *J* =15.6 Hz), 6.51-6.59 (m, 1H); ^{13}C NMR (50 MHz, $CDCl_3$) δ = 3.7, 6.0, 14.6, 21.1, 30.0, 37.0, 59.4, 60.7, 61.0, 87.1, 125.3, 125.9, 141.3, 152.4, 166.0, 169.0; MS (ESI): 334.0 $(M+Na)^+$; Anal. Calcd for $C_{15}H_{21}NO_6$: C, 57.87; H, 6.80; N, 4.50, Found: C, 57.90; H, 6.81; N, 4.52.

(*Z*) 4-(1-Isobutyl-2-hydroxy-3,4-dimethoxy-5-oxo-2,5-dihydro-1*H*-pyrrol-2-yl)-but-2-enoic acid ethyl ester (15): It was obtained by the reaction of **9** (0.6 g, 2.36 mmol) and isobutyl amine (0.24 mL, 2.36 mmol) as above. Colourless oil, yield (0.43 g, 65%); IR (neat): 3385, 1683 cm^{-1}; ^{1}H NMR (200 MHz, $CDCl_3$) δ = 0.89 (d, *J* = 6.6 Hz, 6H), 1.26 (t, *J* = 7.2 Hz, 3H), 1.60 (bs, 1H), 1.96-2.03 (m, 1H), 2.71-2.95 (m, 3H), 3.17-3.27 (m, 1H), 3.78 (s, 3H), 4.07(s, 3H), 4.17 (q, *J* = 7.1 Hz, 2H), 5.81 (d, *J* = 15.6 Hz, 1H), 6.48-6.56 ((m, 1H); ^{13}C NMR (50 MHz, $CDCl_3$) δ = 14.5, 20.9, 28.8, 37.2, 45.9, 59.4, 60.6, 60.9, 86.4, 125.3, 126.1, 141.1, 152.0, 166.0, 168.3; MS (FAB): 328 $(M+H)^+$; Anal. Calcd for $C_{16}H_{25}NO_6$: C, 58.70; H, 7.70; N, 4.28, Found: C, 58.72; H, 7.71; N, 4.29

(*Z*) 4-(1-Hexyl-2-hydroxy-3,4-dimethoxy-5-oxo-2,5-dihydro-1*H*-pyrrol-2-yl)-but-2-enoic acid ethyl ester (16): It was obtained by the reaction of **9** (1.1 g, 4.33 mmol) and

hexyl amine (0.57 mL, 4.33 mmol) as above. Colourless oil (1.2 g, 77%); IR (neat): 3353, 1682, 1596 cm^{-1}; ^{1}H NMR (200 MHz, $CDCl_3$) δ = 0.88 (t, *J* =7.0 Hz , 3H), 1.23-1.30 (m, 7H), 1.46-1.57 (m , 3H), 2.71-2.80 (m, 2H), 3.11-3.30 (m, 4H), 3.78 (s, 3H), 4.06 (s, 3H), 4.14 (q, *J* = 7.0 Hz, 2H), 5.89 (d, *J* = 15.5 Hz, 1H), 6.46-6.61 (m, 1H); ^{13}C NMR (50 MHz, $CDCl_3$) δ = 14.4, 14.5, 22.9, 27.3, 29.7, 29.9, 31.8, 38.5, 59.3, 60.6, 60.9, 86.1, 125.3, 126.1, 141.1, 152.1, 167.1, 169.7; MS (ESI): 356 $(M+H)^+$; Anal. Calcd for $C_{18}H_{29}NO_6$: C, 60.83; H, 8.22; N, 3.94, Found: C, 60.85; H, 8.23; N, 3.94.

(*Z*) 4-(1-Octyl-2-hydroxy-3,4-dimethoxy-5-oxo-2,5-dihydro-1*H*-pyrrol-2-yl)-but-2-enoic acid ethyl ester (17): It was obtained by the reaction of **9** (0.8 g, 3.14 mmol) and octyl amine (0.24 mL, 3.14 mmol) and purified as above. Colourless oil (0.88 g, 73%); IR (neat): 3366, 2930, 1686 cm^{-1}; ^{1}H NMR (200 MHz, $CDCl_3$) δ = 0.87 (t, *J* = 6.7 Hz, 3H), 1.20-1.30 (m, 11H), 1.35-1.60 (m, 3H), 2.74-2.82 (m, 2H), 2.93-3.32 (m, 4H), 3.79 (s, 3H), 4.06 (s, 3H), 4.14 (q, *J* = 7.1 Hz, 2H), 5.90 (d, J= 15.6, 1H), 6.37-6.62 (m, 1H); ^{13}C NMR (50 MHz, $CDCl_3$) δ = 14.4, 14.5, 22.9, 27.7, 29.6, 30.0, 32.1, 37.2, 38.6, 59.4, 60.7, 61.0, 86.3, 125.4, 126.1, 141.0, 152.1, 166.2, 170.1; MS (ESI): 406 $(M+Na)^+$; Anal. Calcd for $C_{20}H_{33}NO_6$: C, 62.64; H, 8.67; N, 3.65, Found: C, 62.66; H, 8.68; N, 3.64.

(*Z*) 4-(1-Propyl-2-hydroxy-3,4-dimethoxy-5-oxo-2,5-dihydro-1*H*-pyrrol-2-yl)-but-2-enoic acid ethyl ester (18): It was obtained by the reaction of **9** (0.7 g, 2.75 mmol) and propylamine (0.23 mL, 2.75 mmol). Colorless oil (0.7 g, 81%); IR (neat): 3329, 2974, 1687 cm^{-1}; ^{1}H NMR (300 MHz, $CDCl_3$) δ = 093 (t, *J* =7.3 Hz, 3H), 1.28 (t, *J* = 7.1 Hz, 3H), 1.57-1.73 (m, 3H) 2.75-2.85 (m, 2H), 3.05-3.15 (m,1H), 3.31-3.41 (m,1H), 3.83 (s, 3H), 4.07 (s, 3H), 4.15 (q, *J* = 7.1 Hz, 2H), 5.86 (d, *J* = 15 Hz, 1H), 6.54-6.59 (m, 1H); ^{13}C NMR (50 MHz, $CDCl_3$) δ = 12.0, 15.5, 23.0, 39.4, 40.5, 59.4, 60.9, 61.9, 85.1, 125.5, 126.2, 141.0, 153.5, 166.2, 167.8; MS (ESI): 336 $(M+Na)^+$; Anal. Calcd for $C_{15}H_{23}NO_6$: C, 57.50; H, 7.40; N, 4.47, Found: C, 57.49; H, 7.43; N, 4.49.

(*Z*) 4-(2-hydroxy-3,4-dibenzyloxy-5-oxo-2,5-dihydro-1*H*-pyrrol-2-yl)-but-2-enoic acid ethyl ester (19): It was obtained by the reaction of **10** (0.65 g, 1.6 mmol) and

ethanolic ammonia (10 mL) as colourless gum, yield (0.58 g, 86%). IR (neat): 3310, 1709, 1680 cm^{-1}; ^{1}H NMR (200 MHz, $CDCl_3$): δ 1.26 (t, *J* = 7.2 Hz, 3H), 2.54-2.74 (m, 2H), 4.14 (q, *J* = 7.1 Hz, 2H), 4.92 (d, *J* = 12.8 Hz, 2H), 5.07 (d, *J* = 12.4 Hz, 2H), 5.84 (d, *J* = 15.5 Hz, 1H), 6.76-6.86 (m, 1H), 7.17-7.32 (m, 10H); ^{13}C NMR (50 MHz, $CDCl_3$) δ = 14.6, 30.0, 37.0, 39.5, 60.7, 73.2, 74.9, 93.6, 123.5, 125.9, 127.9, 128.5, 128.8, 128.9, 129.4, 136.5, 136.7, 141.7, 154.8, 166.4, 170.3; MS (ESI): 446 (M+Na)$^{+}$; Anal. Calcd. for $C_{24}H_{25}NO_6$: C, 68.07; H, 5.95; N, 3.31, Found: C, 68.09; H, 5.96; N, 3.33.

(*Z*) 4-(1-Butyl-2-hydroxy-3,4-dibenzyloxy-5-oxo-2,5-dihydro-1*H*-pyrrol-2-yl)-but-2-enoic acid ethyl ester (20): It was obtained by the reaction of **10** (0.5 g, 1.24 mmol) and butyl amine (0.12 mL, 1.24 mmol) as colourless granules, yield (0.43 g, 73.5%); m.p. 50-52 ºC; IR (neat): 3277, 1723, 1675, 1598 cm^{-1}; ^{1}H NMR (200 MHz, $CDCl_3$) δ = 0.94 (t, *J* = 7.1 Hz, 3H), 1.25-1.40 (m, 5H), 1.56-1.67 (m, 2H), 2.70-2.77 (m, 2H), 2.94 (s, 1H), 3.15-3.38 (m, 2H), 4.16 (q, *J* = 7.0 Hz, 2H,), 4.80-5.15 (m, 4H), 5.77 (d, *J* = 15.6 Hz, 1H), 6.40-6.67 (m, 1H), 7.15-7.33 (m, 10H); ^{13}C NMR (50 MHz, $CDCl_3$) δ = 14.1, 14.7, 20.9, 32.1, 37.3, 38.4, 60.7, 73.2, 74.9, 86.6, 124.0, 125.5, 127.9 128.6, 128.8, 128.9, 129.5, 136.5, 136.6, 141.2, 152.2, 166.0, 168.2; MS (ESI): 502 (M+Na)$^{+}$, 480 (M+H)$^{+}$; Anal. Calcd. for $C_{28}H_{33}NO_6$: C, 70.13; H, 6.94; N, 2.93, Found: C, 70.14; H, 6.96; N, 2.94.

(*Z*) 4-(1-Hexyl-2-hydroxy-3,4-dibenzyloxy-5-oxo-2,5-dihydro-1*H*-pyrrol-2-yl)-but-2-enoic acid ethyl ester (21): It was obtained by the reaction of **10** (0.5 g, 1.24 mmol) and hexylamine (0.16 mL, 1.24 mmol) as colourless solid, yield (0.45 g, 72.5%); m.p. 54-55 ºC; IR (neat): 3270, 1670, 1596 cm^{-1}; ^{1}H NMR (200 MHz, $CDCl_3$) δ = 0.88 (t, *J* = 7.1 Hz, 3H), 1.13-1.28 (m, 9H), 1.54-1.60 (m, 2H), 2.69-2.76 (m, 2H), 3.06-3.32 (m, 2H), 4.16 (q, *J* =7.0 Hz, 2H), 4.79-5.15 (m, 4H), 5.77 (d, *J* = 15.5 Hz, 1H), 6.50-6.70 (m, 1H), 7.15-7.33 (m, 10H); ^{13}C NMR (50 MHz, $CDCl_3$) δ = 14.4, 14.6, 23.0, 25.1, 27.4, 30.0, 31.8, 37.3, 136.5, 136.6, 141.1, 152.1, 166.0, 168.1; MS (ESI): 530 (M+Na)$^{+}$; Anal. Calcd. for $C_{30}H_{37}NO_6$: C, 70.97; H, 7.35; N, 2.76, Found: C, 70.99; H, 7.38; N, 2.75.

(*Z*) 4-(1-Benzyl-2-hydroxy-3,4-dibenzyloxy-5-oxo-2,5-dihydro-1*H*-pyrrol-2-yl)-but-2-enoic acid ethyl ester (22):: It was obtained by the reaction of **10** (0.78 g, 1.93 mmol) and benzylamine (0.21 mL, 1.93 mmol) as an off-white solid (0.8 g, 81.6%); m.p. 66-68 ºC; IR (neat): 3423, 1680, 1592 cm^{-1}; ^{1}H NMR (300 MHz, $CDCl_3$) δ = 1.25 (t, *J* = 7.1 Hz, 3H, CH_3), 2.50-2.65 (m, 3H, -CH_2, OH), 4.09 (q, 2H, *J* = 7 Hz) 4.28-4.39 (m, 2H), 5.03-5.23 (m, 4H), 5.33 (d, *J* = 15.6 Hz, 1H), 6.23-6.31 (m, 1H), 7.10-7.34 (m,15H); ^{13}C NMR (50 MHz, $CDCl_3$) δ = 14.7, 37.5, 41.5, 60.4, 73.5, 75.1, 87.1, 123.9, 125.2, 127.2, 127.5, 127.9, 128.2, 128.6, 128.8, 129.0, 129.3, 129.6, 136.4, 136.5, 138.8, 140.7, 153.1, 175.9, 168.7; MS (ESI): 536 $(M+Na)^+$; Anal. Calcd for $C_{31}H_{31}NO_6$: C, 72.50; H, 6.08; N, 2.73, Found: C, 72.52; H, 6.11; N, 2.76.

4-(3,4-Dimethoxy-5-oxo-1,5-dihydro-pyrrol-2-ylidene)-but-2-enoic acid ethyl ester (23): General Procedure: A solution of the above compound **11** (0.7 g, 2.58 mmol) in anh. CH_2Cl_2 (5 mL) and *p*-toluene sulphonic acid (*p*TSA, 0.49g, 2.58 mmol) was stirred magnetically for 30 min. at 30 ºC till the disappearance of the starting material (TLC). The reaction mixture was neutralized (pH 7.0) with solid $NaHCO_3$, filtered and filtrate was concentrated to give a crude mass. The latter was dissolved in ethyl acetate (100 mL), washed with water (2 x 25 mL), the organic layer dried (Na_2SO_4) and evaporated under reduced pressure to give a gummy mass. The latter was chromatographed over silica gel (60-120 mess) using a gradient of (1:19 → 1:4) as eluent to give compound **23** as colourless solid. Yield (0.450 g, 68.9%), m.p. 167-169 ºC; IR (neat): 1719, 1607 cm^{-1}; ^{1}H NMR (300 MHz, $CDCl_3$) δ = 1.32 (t, *J* = 7.1 Hz, 3H, CH_3), 4.00 (*s*, 3H, OCH_3), 4.02 (*s*, 3H, OCH_3), 4.10 (q, *J* = 7.0 Hz, 2H, OCH_2), 5.93 (d, *J* = 12.3 Hz, 1H, H-6), 5.96 (d, *J* = 15.0 Hz, 1H, H-8), 7.75 (dd, *J* = 12.4 Hz, *J* = 12.5 Hz,1H, H-7), 9.13 (s, 1H, NH). ^{13}C NMR (50 MHz, $CDCl_3$) δ = 14.6, 59.7, 60.8, 61.2, 105.1, 122.4, 123.3, 129.5, 136.9, 137.9, 144.6, 167.2, 167.7; MS (ESI): 254 $(M+H)^+$; Anal. Calcd. for $C_{12}H_{15}NO_5$: C, 56.91; H, 5.97; N, 5.53, Found: C, 56.92; H, 5.99; N, 5.54.

4-(1-Benzyl-3,4-dimethoxy-5-oxo-1,5-dihydro-pyrrol-2-ylidene)-but-2-enoic acid ethyl ester (24): It was obtained by the reaction of the above compound **12** (0.65 g, 1.8 mmol) and *p*-TSA (0.34 g, 1.8 mmol) as above. Colourless foam (0.4 g, 65.5%), IR

(neat): 1704, 1625 cm^{-1}; ^{1}H NMR (300 MHz, $CDCl_3$) δ = 1.28 (t, *J* = 7.1 Hz, 3H), 4.02 (s, 3H), 4.17 (s, 3H), 4.21(q, *J* = 7.1 Hz, 2H), 4,79 (s, 2H), 5.80 (d, *J* = 15.3 Hz, 1H), 5.85 (d, *J* =11.7 Hz, 1H), 7.18-7.36 (m, 5H), 8.06 (dd, J=12.0 Hz ,1H); ^{13}C NMR (50 MHz, $CDCl_3$): δ = 14.7, 42.4, 60.0, 60.5, 60.8,109.5, 110.2, 110.6, 123.7,127.0 (2C), 127.8, 29.1 (2C), 136.9, 139.5, 145.3, 165.1, 167.0; MS(ESI): 344.1 $(M+H)^+$; Anal. Calcd for $C_{19}H_{21}NO_5$: C, 66.46; H, 6.16; N, 4.08, Found: C, 66.47; H, 6.19; N, 4.10.

4-(1-Butyl-3,4-dimethoxy-5-oxo-1,5-dihydro-pyrrol-2-ylidene)-but-2-enoic acid ethyl ester (25): It was obtained by the reaction of **13** (0.6 g, 1.83 mmol) and *p*-TSA (0.34 g, 1.83 mmol) as an oil (0.4 g, 71%). IR (neat): 1700, 1620, 1259 cm^{-1}; ^{1}H NMR (200 MHz, $CDCl_3$) δ = 0.91 (t, *J* = 7.1 Hz, 3H), 1.30 (t, *J* = 7.1 Hz, 3H), 1.38-1.56 (m, 4H), 3.53 (t, *J* = 6.9 Hz, 2H), 3.96 (s, 3H), 4.13 (s, 3H), 4.23 (q, *J* = 7.0 Hz, 2H), 5.83 (d, *J* = 12.0 Hz, 1H) 5.89 (d, *J* = 15.2 Hz, 1H), 8.12 (dd, J= 12.0 Hz,1H); ^{13}C NMR (50 MHz, $CDCl_3$) δ = 14.1, 14.7, 20.4, 30.0, 31.1, 38.5, 40.7, 59.9, 60.5, 60.6, 104.3, 108.8, 123.1, 129.9, 137.3, 139.8, 144.8, 164.9, 167.1; MS (ESI): 310 $(M+H)^+$; Anal. Calcd for $C_{16}H_{23}NO_5$: C, 62.12; H, 7.49; N, 4.53, Found: C, 62.15; H, 7.46; N, 4.54.

4-(1-Cyclopropyl-3,4-dimethoxy-5-oxo-1,5-dihydro-pyrrol-2-ylidene)-but-2-enoic acid ethyl ester (26): It was obtained by the reaction of the above compound **14** (0.54 g, 1.73 mmol) and *p*-TSA (0.32 g, 1.73 mmol) as colourless solid. Yield (0.31 g, 62%); m.p. 62-64 ºC; IR (neat): 1700, 1620, 1354 cm^{-1}; ^{1}H NMR (200 MHz, $CDCl_3$) δ = 0.87-0.99 (m, 4H), 1.31 (t, *J* = 7.0 Hz, 3H), 2.73 (m, 1H), 3.93 (s, 3H), 4.12(s, 3H), 4.23 (q, *J* = 7.0 Hz, 2H), 5.90 (d, *J* = 15.3 Hz, 1H), 6.26 (d, *J* = 12.2 Hz, 1H), 8.07 (dd, *J* = 12.3 Hz, *J* = 12.4Hz, 1H,); ^{13}C NMR (50 MHz, $CDCl_3$) δ = 6.7, 14.6, 21.1, 60.0, 60.6, 60.8, 110.1, 123.4, 129.4, 138.5, 139.8, 144.8, 165.6, 167.3; MS (ESI): 294 $(M+H)^+$; Anal. Calcd for $C_{15}H_{19}NO_5$: C, 61.42; H, 6.53; N, 4.78, Found: C, 61.44; H, 6.56; N, 4.76.

4-(1-Isobutyl-3,4-dimethoxy-5-oxo-1,5-dihydro-pyrrol-2-ylidene)-but-2-enoic acid ethyl ester (27): It was obtained by the reaction of the above compound **15** (0.55 g, 1.68 mmol) and *p*-TSA (0.31 g, 1.68 mmol) as colourless granules. Yield (0.3 g, 58%), m.p: 56-58 ºC; IR (neat): 1680, 1619 cm^{-1}; ^{1}H NMR (300MHz, $CDCl_3$) δ = 0.90 (d, *J* = 6.7 Hz,

6H), 1.31 (t, J = 7.1 Hz, 3H), 1.84-2.1 (m, 1H), 3.36(d, J = 7.5 Hz, 2H), 3.98 (s, 3H), 4.14(s, 3H), 4.19 (q, J = 7.1Hz, 2H), 5.87 (d, J = 12 Hz, 1H), 5.92 (d, J = 15.3 Hz, 1H), 8.16 (dd, J = 12 Hz, 1H); ^{13}C NMR (50 MHz, $CDCl_3$) δ = 14.7, 20.4, 28.3, 46.1, 59.9, 60.5, 60.6, 109.1, 123.1, 129.7, 137.7, 139.7, 144.8, 165.1, 167.0; MS (ESI): 310 $(M+H)^+$; Anal. Calcd for $C_{16}H_{23}NO_5$: C, 62.12; H, 7.49; N, 4.53, Found: C, 62.13; H, 7.52; N, 4.55.

4-(1-Hexyl-3,4-dimethoxy-5-oxo-1,5-dihydro-pyrrol-2-ylidene)-but-2-enoic acid ethyl ester (28): It was obtained by the reaction of compound **16** (0.4 g, 1.12 mmol) and *p*-TSA (0.21 g, 1.12 mmol) as colourless oil, yield (0.19 g, 55%); IR (neat): 1700, 1620 cm^{-1}; ^{1}H NMR (200 MHz, $CDCl_3$) δ = 0.88 (t, J = 7.0 Hz, 3H), 1.29 (m, 7H), 1.52-1.63 (m, 4H), 3.41-3.55 (m, 2H), 3.96 (s, 3H), 4.13 (s, 3H), 4.23 (q, J = 6.9 Hz, 2H), 5.83 (d, J = 12.0 Hz, 1H), 5.88 (d, J = 14.6 Hz, 1H), 8.09 (dd, J = 12.2 Hz, 1H); ^{13}C NMR (50 MHz, $CDCl_3$) δ = 14.3, 14.6, 22.9, 26.8, 28.9, 31.8, 38.8, 60.0, 60.7, 60.9, 109.0, 123.2, 130.0, 137.3, 139.8, 145.0, 165.1, 167.4; MS (ESI): 338.2 $(M+H)^+$; Anal. Calcd for $C_{18}H_{27}NO_5$: C, 64.07; H, 8.07; N, 4.15, Found: C, 64.09; H, 8.10; N, 4.16.

4-(1-Octyl-3,4-dimethoxy-5-oxo-1,5-dihydro-pyrrol-2-ylidene)-but-2-enoic acid ethyl ester (29): It was obtained by the reaction of the above compound **17** (0.4 g, 1.04 mmol) and *p*-TSA (0.19 g, 1.04 mmol) as above. Colourless oil, yield (0.23 g, 60.5%); IR (neat): 1705, 1618 cm^{-1}; ^{1}H NMR (300 MHz, $CDCl_3$) δ = 0.88 (t, J = 6.9 Hz, 3H), 1.27-1.55(m, 15H), 3.46-3.59 (m, 2H), 3.86 (s, 3H), 4.12 (s, 3H), 4.19 (q, J = 7.2 Hz, 2H), 5.88 (d, J = 12 Hz, 1H), 5.93 (d, J = 15.3 Hz, 1H), 8.16 (dd, J = 12.0 Hz, 12.3 Hz, 1H); ^{13}C NMR (50 MHz, $CDCl_3$) δ = 14.4, 14.7, 22.9, 27.1, 29.0, 29.5, 32.1, 38.7, 59.9, 60.5, 60.6, 108.8, 123.1, 129.9, 137.3, 139.8, 144.8, 164.8, 167.1. MS (ESI): 366.2 $(M+H)^+$; Anal. Calcd for $C_{20}H_{31}NO_5$: C, 65.73; H, 8.55; N, 3.83, Found: C, 65.71; H, 8.59; N, 3.86.

4-(1-Propyl-3,4-dimethoxy-5-oxo-1,5-dihydro-pyrrol-2-ylidene)-but-2-enoic acid ethyl ester (30): It was obtained by the reaction of compound **18** (0.31 g, 0.99 mmol) and *p*-TSA (0.18 g, 0.99 mmol) as described above for compound **17**. Colourless oil, yield (0.18 g, 62%); IR (neat): 1702, 1620, 1219 cm^{-1}; ^{1}H NMR (200 MHz, $CDCl_3$) δ = 0.91 (t,

J = 7.1 Hz, 3H), 1.30 (t, J = 7.0 Hz, 3H), 1.51-1.66 (m, 2H), 3.47-3.55 (m, 2H), 3.96 (s, 3H), 4.13 (s, 3H), 4.20 (q, J = 7.0 Hz, 2H), 5.86 (d, J = 8.6 Hz, 1H), 5.92(d, J = 11.7 Hz, 1H), 8.12 (dd, J = 12.0 Hz, J = 12.8 Hz, 1H); ^{13}C NMR (50 MHz, $CDCl_3$) δ = 11.5, 14.5, 22.2, 40.3, 60.0, 60.7, 60.9, 109.2, 123.3, 137.3, 139.8, 165.2, 167.4; MS (ESI): 296 $(M+H)^+$; Anal. Calcd for $C_{15}H_{21}NO_5$: C, 61.00; H, 7.17; N, 4.74, Found: C, 72.84; H, 7.20; N, 4.76.

4-(3, 4-Dibenzyloxy-5-oxo-1, 5-dihydro-pyrrol-2-ylidene)-but-2-enoic acid ethyl ester (31): It was obtained by the reaction of the above compound **19** (0.6g, 1.41 mmol) and *p*-toluene sulphonic acid (0.26 g, 1.41 mmol) as usual. White granules, yield (0.36 g, 63.1%); m.p: 101-103 ºC; IR (neat): 3427, 2364, 1625 cm^{-1}; ^{1}H NMR (300 MHz, $CDCl_3$) δ = 1.31 (t, J = 7.1 Hz, 3H), 4.18 (q, J = 7.1Hz, 2H), 5.22 (s, 2H), 5.33 (s, 2H), 5.95 (d, J = 14.0 Hz, 2H), 7.25-7.38 (m, 10H), 7.79 (dd, J = 13.6 Hz, J = 12.4 Hz, 1H), 9.29 (s, 1H); ^{13}C NMR (50 MHz, $CDCl_3$) δ = 14.6, 61.2, 73.5, 74.4, 105.2, 122.6, 128.1 (3C), 128.9 (3C), 129.2 (3C), 136.7, 137.0, 137.7, 144.2, 167.2, 167.7; MS (ESI): 406 $(M+H)^+$; Anal. Calcd for $C_{24}H_{23}NO_5$: C, 71.10; H, 5.72; N, 3.45, Found: C, 71.12; H, 5.73; N, 3.48.

4-(1-Butyl-3,4-dibenzyloxy-5-oxo-1,5-dihydro-pyrrol-2-ylidene)-but-2-enoic acid ethyl ester (32): It was obtained by the reaction of compound **20** (0.7 g, 1.46 mmol) and *p*-TSA (0.27 g, 1.46 mmol) as colourless oil, yield (0.37 g, 55.2%). IR (neat): 2960, 2362, 1599 cm^{-1}. ^{1}H NMR (300 MHz, $CDCl_3$) δ = 0.92 (t, J= 7.2 Hz, 3H), 1.18 (t, J= 7.2 Hz, 3H,), 1.23-1.38 (m, 2H), 1.47-1.57 (m, 2H), 3.56 (t, J = 7.2 Hz, 2H), 4.18 (q, J = 7.2 Hz, 2H), 5.27 (s, 4H), 5.85 (d, J = 12.0 Hz, 1H), 5.91 (d, J = 15.3 Hz, 1H), 8.18 (dd, J = 12.0 Hz, J = 12.3 Hz, 1H); ^{13}C NMR (75 MHz, $CDCl_3$) δ = 13.7, 14.3, 20.0, 30.7, 38.1, 60.1, 73.6, 73.9, 108.8, 123.1, 127.6, 128.2, 128.5, 128.5, 128.7, 136.1 136.4, 136.9, 139.2, 143.8, 164.6, 166.5; MS (ESI): 462 $(M+H)^+$; Anal. Calcd. for $C_{28}H_{31}NO_5$: C, 72.86; H, 6.77; N, 3.03, Found: C, 72.84; H, 6.80; N, 3.05.

4-(1-Hexyl-3,4-dibenzyloxy-5-oxo-1,5-dihydro-pyrrol-2-ylidene)-but-2-enoic acid ethyl ester (33): It was obtained by the reaction of the above compound **21** (0.31 g, 0.61 mmol) and *p*-TSA (0.11 g, 0.61 mmol) as colourless foam (0.18 g, 62%); IR (neat): 1699,

1620 cm^{-1}; ^{1}H NMR (300 MHz, $CDCl_3$) δ = 0.92 (t, *J* = 6.5 Hz, 3H), 1.21 (t, Hz = 7.1 Hz, 3H), 1.27-1.58 (m, 8H), 3.57 (t, Hz = 7.1 Hz, 2H), 4.11 (q, J= 7.1 Hz, 2H), 5.29 (s, 4H), 5.86 (d, *J* = 12 Hz, 1H), 5.92 (d, *J* = 15 Hz, 1H), 7.27-7.41 (m, 10H), 8.19 (dd, *J* = 12.1 Hz, 1H); ^{13}C NMR (50 MHz, $CDCl_3$) δ = 14.4, 14.7, 22.9, 26.8, 29.0, 31.8, 38.8, 60.4, 74.0, 74.3, 109.1, 123.5, 128.0, 128.6, 128.9, 129.1, 136.5, 136.8, 137.3, 139.7, 144.2, 164.9, 166.8; MS (ESI): 490 $(M+H)^+$; Anal. Calcd for $C_{30}H_{35}NO_5$: C, 73.59; H, 7.21; N, 2.86, Found: C, 73.62; H, 7.24; N, 2.88.

4-(1-Benzyl-3,4-dibenzyloxy-5-oxo-1,5-dihydro-pyrrol-2-ylidene)-but-2-enoic acid ethyl ester (34): It was obtained by the reaction of the above compound **22** (0.44 g, 0.85 mmol) and *p*-TSA (0.16 g, 0.85 mmol) as colourless granules (0.22 g, 55%); IR (neat): 2370, 1700, 1620 cm^{-1}; ^{1}H NMR (300 MHz, $CDCl_3$) δ = 1.18 (t, *J* = 7.2 Hz, 3H), 4.14 (q, *J* = 7.2 Hz, 2H), 5.07-5.37 (m, 6H), 5.75 (d, *J* = 15 Hz, 1H), 5.81 (d, *J* = 12 Hz, 1H), 7.16-7.46 (m, 15H), 8.12 (dd, *J* = 12.0 Hz, 1H); ^{13}C NMR (50 MHz, $CDCl_3$) δ = 14.6, 42.5, 60.5, 74.1, 74.5, 110.5, 124.0, 127.0, 127.9, 128.1, 128.7, 129.0, 129.2, 136.4, 136.9, 139.3, 144.8, 165.3, 166.9; MS (ESI): 496 $(M+H)^+$; Anal. Calcd for $C_{31}H_{29}NO_5$: C, 75.13; H, 5.90; N, 2.83, Found: C, 75.16; H, 5.92; N, 2.82.
